# Supplementary material for: The Evolution and Characterization of the RNA Interference Pathways in Lophotrochozoa
Source: Genome Biol Evol. 2024 May 7;16(5):evae098. doi: 10.1093/gbe/evae098 (PMC11114477; doi:10.1093/gbe/evae098)
Supplement: evae098_Supplementary_Data [file evae098_supplementary_data.zip › Supplementary_materials.zip!/selected_miRNAs/NoGe_selected_miRNAs/known_miRNA_families_pdf_files/NMRB01001187.1_Notospermus_geniculatus_strain_Ushimado_scaffold1187,_whole_genome_shotgun_sequence_26007.pdf]

Provisional ID	:	NMRB01001187.1_Notospermus_geniculatus_strain_Ushimado_scaffold1187,_whole_genome_shotgun_sequence_26007
Score total	:	2368407.3
Score for star read(s)	:	3.9
Score for read counts	:	2368398.9
Score for mfe	:	1.6
Score for randfold	:	0
Score for cons. seed	:	3
Total read count	:	4645524
Mature read count	:	4645442
Loop read count	:	0
Star read count	:	82

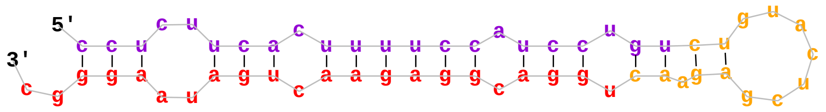

Genome coordinates: NMRB01001187.1 152626-152684

pre-miRNA sequence:
CCUCUUCACUUUUCCAUCCUGUCCUCUUCACUUUUCCAUCCUGUCUGUACUCGAGAACUGGACGGAGAACUGAUAAGGGC

Mature sequence: UGGACGGAGAACUGAUAAGGGC

Aligned mature sequence of the *N. geniculatus* pre-miRNA with the top 5 hits from the BLAST search against the pre-miRNA mirGeneDB set:

```
                            **********************
Nge_Mir-184                 uggacggagaacugauaagggc
Obi-Mir-184-P26_3p          uggacggagaacugauaagggc
Ovu-Mir-184-P26_3p          uggacggagaacugauaagggc
Esc-Mir-184_3p              uggacggagaacugauaagggc
Npo-Mir-184_3p              uggacggagaacugauaagggc
Lan-Mir-184_3p              uggacggagaacugauaagggc
```
